# Triple therapy approach for treating chromoblastomycosis in a Lebanese patient

**DOI:** 10.1002/ccr3.9392

**Published:** 2024-09-30

**Authors:** Joe Khodeir, Paul Ohanian, Hala Abi Rached Megarbane

**Affiliations:** ^1^ Department of Dermatology at Saint Georges Hospital University Medical Center, Faculty of Medicine and Medical Sciences University of Balamand Beirut Lebanon; ^2^ Department of Family Medicine at Saint Georges Hospital University Medical Center, Faculty of Medicine and Medical Sciences University of Balamand Beirut Lebanon; ^3^ Department of Dermatology at Saint Georges Hospital University Medical Center, Faculty of Medicine and Medical Sciences Saint George University of Beirut Beirut Lebanon

**Keywords:** chromoblastomycosis, dermatological treatment, fungal infection, infectious dermatology, tropical skin infection

## Abstract

Chromoblastomycosis, though rare in non‐endemic regions like Lebanon, should be considered in patients presenting with chronic, verrucous skin lesions unresponsive to conventional therapies. Multimodal treatment combining oral antifungals, cryotherapy, and adjunctive topical 5‐Fluorouracil demonstrates efficacy in managing refractory cases. Follow‐up visits three and 6 months after treatment cessation showed sustained lesion clearance and no recurrence.

## 1 INTRODUCTION

Chromoblastomycosis is a chronic granulomatous infection of the skin caused by several pigmented fungi, resulting in the formation of slow‐growing verrucous plaques and nodules.^1^ This condition is predominantly seen in tropical and subtropical regions, with *Fonsecaea* spp. and *Cladophialophora* spp. being the most common causative agents. The infection typically arises following traumatic inoculation of the fungi through minor injuries often associated with agricultural activities.^2^ The disease is characterized by its slow progression and potential to mimic various dermatological conditions, which can complicate diagnosis and delay appropriate treatment.^1^ This article presents the first documented case of chromoblastomycosis in Lebanon,^3^ highlighting the need for awareness and consideration of this diagnosis even in non‐endemic regions.

## 2 CASE HISTORY/EXAMINATION

**FIGURE 1:**
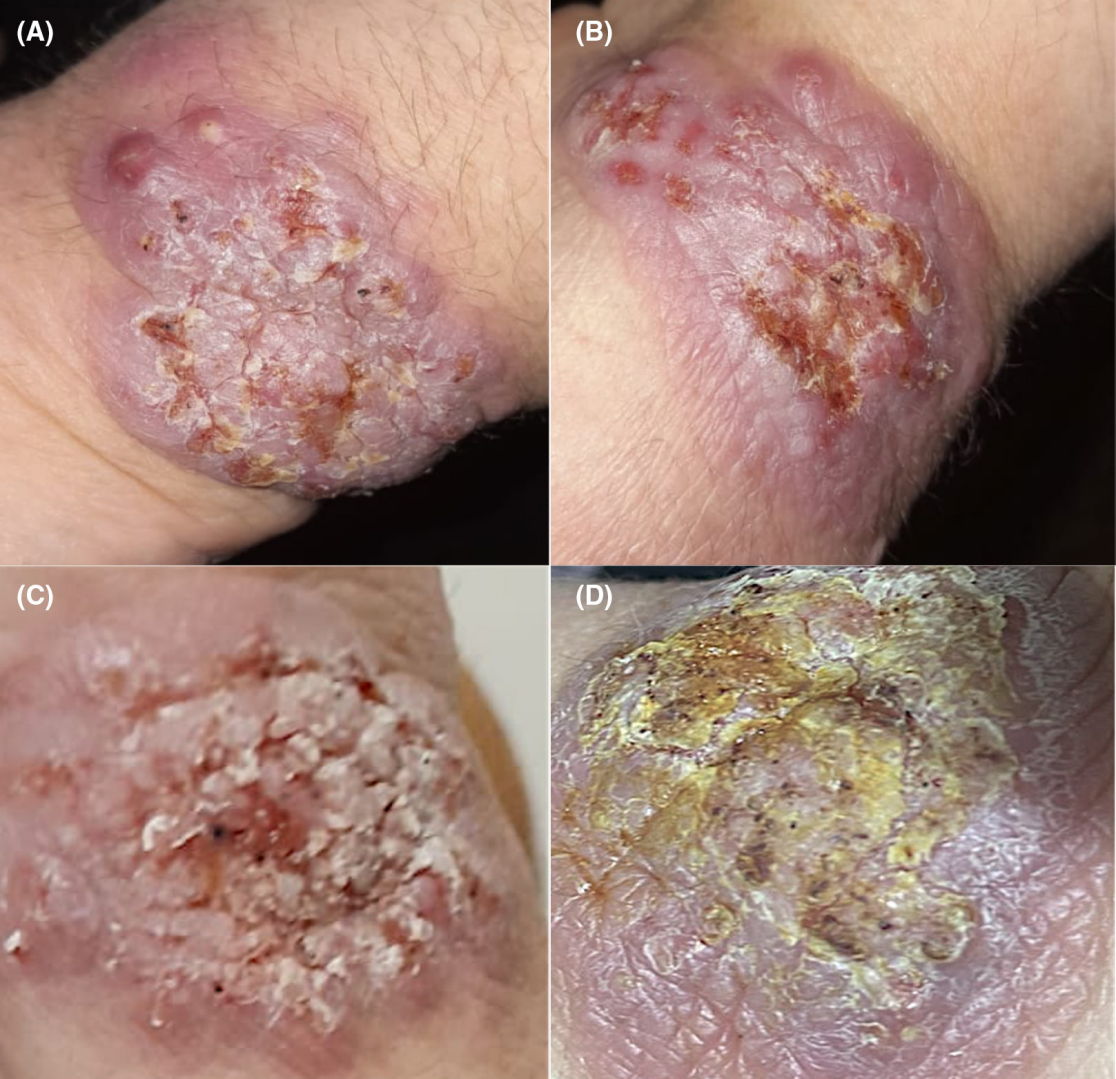
(A, B) Hyperkeratotic erythematous nodular lesions with secondary ulceration and pustules. (C) Four weeks after initial presentation, the lesion became more hyperkeratotic and exophytic in appearance, with overlying (D) black dots on the surface of the lesion, representing sclerotic bodies.

A 70‐year‐old female with diabetes and heart failure presented with asymptomatic plaques and nodules on her left hand over 6 months, treated unsuccessfully with a 2 weeks course of 60 mg oral prednisone daily, clindamycin 900 mg daily and ciprofloxacin 500 mg twice daily. The patient denied any associated systemic signs including fever, cough, dyspnea, or other respiratory complaints. Physical examination revealed three cauliflower‐like hyperkeratotic nodules with secondary ulcerations and pustules on the left forearm, without lymphadenopathy (Figure 1a,b). She denied any insect bites, occupational exposure, or travel history, but reported spending time gardening.

## 3 METHODS

### 3.1 Differential Diagnosis, Investigations, and Treatment

The differential diagnoses included tuberculous and non‐tuberculous mycobacterial skin infection, neutrophilic dermatosis, sarcoidosis, deep fungal infection of the skin, sporotrichiosis, and leishmaniasis. Multiple biopsies were taken for histopathologic examination, mycobacterial PCR analysis, acid‐fast culture, bacterial, and fungal cultures. Chest X‐ray and a PPD skin test were performed. Both tests were negative, showing no pulmonary abnormalities or evidence of latent tuberculosis infection.

**FIGURE 2:**
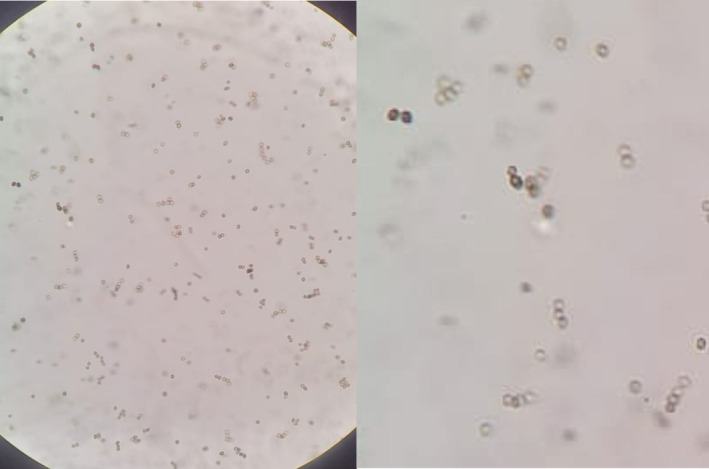
Numerous muriform/sclerotcic bodies seen on KOH examination.

Histopathology showed hyperkeratotic skin with parakeratosis, pseudo‐epithiliomatous epidermis and marked mixed interstitial inflammation with granulomatous and abscess formation in the dermis in favor of an infectious process. Special stains, mycobacterial PCR and acid fast cultures were negative. Bacterial culture was positive for Streptococcus agalactiae and the patient was started on amoxicillin/clavulanic acid for a duration of 2 weeks. However, 4 weeks later, lesions kept growing and became more exophytic and verrucous in appearance with overlying black dots (Figure 1c,d). A deep fungal infection of the skin was highly suspected, specifically chromoblastomycosis. Potassium Hydroxide examination of the black dots from the surface of the lesion showed numerous spherical spores, characteristic of muriform bodies (Figure 2) and fungal culture on sabouraud dextrose agar grew brownish colonies confirming chromoblastomycosis. The patient was started on oral terbinafine 500 mg daily and once weekly sessions of cryotherapy without improvement after 6 weeks, then switched to oral itraconazole at 200 mg daily with no improvemen after 4 weeks.

## 4 RESULTS

**FIGURE 3:**
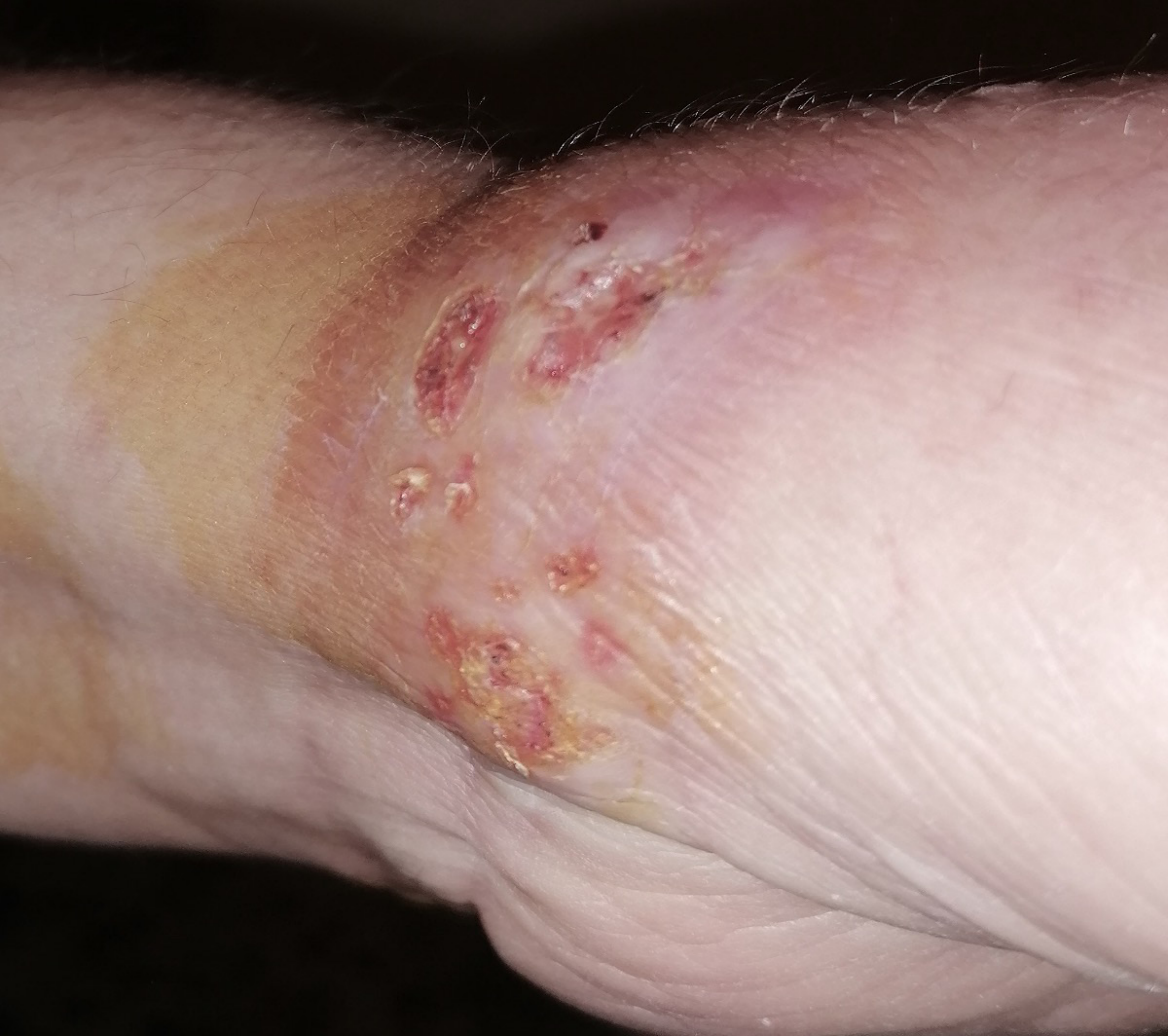
Three months after triple therapy lesions improved drastically.

After two therapy regimens failure, she was initiated on a combination of triple therapy consisting of six sessions of cryotherapy using liquid nitrogen once weekly, and oral itraconazole 100 mg twice daily with topical 5‐Fluorouracil (5‐FU) five times per week. Six weeks later this multidrug regimen resulted in significant lesion regression and was maintained on this regimen for an additional 6 months. At follow‐up patient shows almost 80% clearance in her lesions (Figure 3). The total duration of treatment was 6 months, with no recurrence noted after 3 months of treatment cessation.

## 5 DISCUSSION

Chromoblastomycosis is a tropical skin infection caused by various dematiaceous fungi, such as *Fonsecaea* spp. and *Cladophialophora* spp. Clinically, it manifests as slow‐growing, warty plaques and nodules that can ulcerate.^1^, ^4^ As a neglected tropical disease, chromoblastomycosis can lead to serious complications, including secondary bacterial infections, chronic lymphedema, deep tissue fibrosis, and squamous cell carcinoma.^5^ In our case, Streptococcus agalactiae was identified as a superinfection, marking the first reported association between these two infectious agents. Diagnosing chromoblastomycosis is challenging due to its ability to mimic various infectious and inflammatory disorders.^4^, ^5^ The fungus induces a granulomatous response in the dermis, with pseudoepitheliomatous hyperplasia of the epidermis. The fungal elements, often visible as sclerotic or muriform bodies, are brown and extruded transepidermally, appearing as black dots on the lesion's surface, which is characteristic of chromoblastomycosis.^4^ Potassium hydroxide examination of these black dots, revealing muriform bodies, provides a simpler, less invasive diagnostic method, especially when these bodies are not seen on histology.^5^

In our case, the patient's underlying conditions, including diabetes and heart failure, likely compromised her immune response, increasing her susceptibility to chromoblastomycosis. Systemic diseases such as diabetes mellitus have been shown to increase the risk of fungal infections due to impaired immune function.^6^, ^7^ In a review by Shenoy et al., 41% of patients with chromoblastomycosis had systemic diseases like diabetes and ischemic heart disease, which underscores the association between compromised immunity and infection risk.^4^ Moreover, the patient received oral steroids (60 mg prednisone daily for 2 weeks) as part of her initial treatment. Corticosteroids can significantly suppress the immune system, especially in elderly, making it more difficult for the body to control and resolve fungal infections.^8^ This immunosuppressive effect likely contributed to the persistence and severity of the chromoblastomycosis, complicating the treatment and management of the infection. The combination of diabetes, heart failure, and steroid use may have created a particularly challenging environment for the resolution of the infection. Other factors such as exposure to environmental fungi through gardening also played a role.

The antifungal drugs of choice are itraconazole or terbinafine, administered alone or together for a year or more. Other treatment options include oral potassium iodide solution, cryotherapy, posaconazole, voriconazole, photodynamic therapy, and excision of solitary lesions. In our case, terbinafine and itraconazole monotherapies were ineffective, and other treatment modalities, such as voriconazole or photodynamic therapy, were unavailable. Due to these challenges, we employed a multimodal treatment approach. In addition to oral itraconazole, we utilized cryotherapy and topical 5‐FU to achieve local destruction of the lesions. Cryotherapy helps to physically destroy the infected tissue, while topical 5‐FU offers a targeted chemotherapeutic effect.^9^, ^10^ This combination was intended to enhance the overall treatment efficacy by addressing the infection both systemically and locally. Newer treatment modalities also include imiquimod, a topical immune response modifier, which has been shown to enhance local immune responses and has been used successfully in some cases of chromoblastomycosis.^11^, ^12^, ^13^ Acitretin, a systemic retinoid, has immunomodulatory and anti‐proliferative properties that can be beneficial in the treatment of chronic fungal infections.^12^, ^14^ These agents could provide additional therapeutic options, especially in cases resistant to conventional antifungal therapies.^12^ To our knowledge, this is the first case of chromoblastomycosis reported in Lebanon,^3^ highlighting a novel therapeutic approach for treatment‐resistant cases where broader‐acting antifungals like voriconazole and posaconazole are not available.

## 6 CONCLUSION

Chromoblastomycosis is a neglected tropical disease presenting as slowly growing verrucous lesions. Dermatologists should consider this diagnosis, even in non‐endemic regions, and recognize the efficacy of a multidrug regimen involving cryotherapy, topical 5‐Fluorouracil, and itraconazole in managing treatment‐resistant cases.

## AUTHOR CONTRIBUTIONS

**Joe Khodeir:** Conceptualization; data curation; formal analysis; funding acquisition; investigation; methodology; resources; writing – original draft. **Paul Ohanian:** Investigation; methodology; writing – original draft. **Hala Abi Rached Megarbane:** Supervision; validation; visualization.

## FUNDING INFORMATION

None.

## CONSENT

Written informed consent was obtained from the patient to publish this report in accordance with the journal's patient consent policy.

## Data Availability

The data used to support the findings of this study are included within the article.
